# Supplementary material for: Maintenance of quiescent oocytes by noradrenergic signals
Source: Nat Commun. 2021 Nov 26;12:6925. doi: 10.1038/s41467-021-26945-x (PMC8626438; doi:10.1038/s41467-021-26945-x)
Supplement: Supplementary file 2 — Reporting Summary [file 41467_2021_26945_MOESM2_ESM.pdf]

nature research

Corresponding author(s): Young-Jai You

Last updated by author(s): Oct 5, 2021

Reporting Summary

Nature Research wishes to improve the reproducibility of the work that we publish. This form provides structure for consistency and transparency in reporting. For further information on Nature Research policies, see our Editorial Policies and the Editorial Policy Checklist.

Statistics

For all statistical analyses, confirm that the following items are present in the figure legend, table legend, main text, or Methods section.

n/a	Confirmed	
[ ]	[x]	The exact sample size (*n*) for each experimental group/condition, given as a discrete number and unit of measurement
[ ]	[x]	A statement on whether measurements were taken from distinct samples or whether the same sample was measured repeatedly
[ ]	[x]	The statistical test(s) used AND whether they are one- or two-sided<br>*Only common tests should be described solely by name; describe more complex techniques in the Methods section.*
[ ]	[x]	A description of all covariates tested
[x]	[ ]	A description of any assumptions or corrections, such as tests of normality and adjustment for multiple comparisons
[ ]	[x]	A full description of the statistical parameters including central tendency (e.g. means) or other basic estimates (e.g. regression coefficient) AND variation (e.g. standard deviation) or associated estimates of uncertainty (e.g. confidence intervals)
[ ]	[x]	For null hypothesis testing, the test statistic (e.g. F, t, r) with confidence intervals, effect sizes, degrees of freedom and P value noted<br>*Give P values as exact values whenever suitable.*
[x]	[ ]	For Bayesian analysis, information on the choice of priors and Markov chain Monte Carlo settings
[x]	[ ]	For hierarchical and complex designs, identification of the appropriate level for tests and full reporting of outcomes
[x]	[ ]	Estimates of effect sizes (e.g. Cohen's *d*, Pearson's *r*), indicating how they were calculated

Our web collection on statistics for biologists contains articles on many of the points above.

Software and code

Policy information about availability of computer code

Data collection	Zeiss Zen Lite 2.1, Zeiss AxioVision 4.8.2
Data analysis	Microsoft Excel (USA), GraphPad Prism version 9.1, version 9.2

For manuscripts utilizing custom algorithms or software that are central to the research but not yet described in published literature, software must be made available to editors and reviewers. We strongly encourage code deposition in a community repository (e.g. GitHub). See the Nature Research guidelines for submitting code & software for further information.

Data

Policy information about availability of data

All manuscripts must include a data availability statement. This statement should provide the following information, where applicable:

- Accession codes, unique identifiers, or web links for publicly available datasets
- A list of figures that have associated raw data
- A description of any restrictions on data availability

Source data are provided with this paper.

Field-specific reporting

Life sciences study design

All studies must disclose on these points even when the disclosure is negative.

Sample size	No sample-size calculation was performed. Sample sizes were practically determined based on the variation occurring within experimental groups.
Data exclusions	No data were excluded.
Replication	All experimental findings reported were reproducible. All experiments were performed at least three times and most experiments were repeated at different days.
Randomization	No specific randomization method was used. All the samples were randomly selected from cultivating containers that randomly distributed in the culture area.
Blinding	Blinding is not applied to the experiments because the each sample group was well defined with distinct phenotypes. The control and test groups were always tested in parallel. In addition, we collected all the

Reporting for specific materials, systems and methods

We require information from authors about some types of materials, experimental systems and methods used in many studies. Here, indicate whether each material, system or method listed is relevant to your study. If you are not sure if a list item applies to your research, read the appropriate section before selecting a response.

Materials & experimental systems

n/a	Involved in the study	
☐	☒	Antibodies
☒	☐	Eukaryotic cell lines
☒	☐	Palaeontology and archaeology
☐	☒	Animals and other organisms
☒	☐	Human research participants
☒	☐	Clinical data
☒	☐	Dual use research of concern

Methods

n/a	Involved in the study	
☒	☐	ChIP-seq
☒	☐	Flow cytometry
☒	☐	MRI-based neuroimaging

Antibodies

Antibodies used	Anti-MAPK-YT (M8159, Sigma), anti-mouse secondary antibody conjugated with Alexa Fluor 488 (ab150113, Abcam)
Validation	The Sigma website: https://www.sigmaaldrich.com/US/en/product/sigma/m8159, Reference: Miller et al 2001. Science 291:2144,<br>The abcam website: https://www.abcam.com/goat-mouse-igg-hl-alexa-fluor-488-ab150113.html

Animals and other organisms

Policy information about studies involving animals; ARRIVE guidelines recommended for reporting animal research

Laboratory animals	Caenorhabditis elegans: hermaphrodite and female, L4 and adults<br>Caenorhabditis remanei : female, L4 and adults<br>Drosophila melanogaster: Female 0 day - 2 day of post-eclosion were used for study<br>Danio rerio strains used were dbhct806/+ (AB/TL) and Tg(dbh:EGFP). 3 months - 6 months old females were used.
Wild animals	No wild animals were used in the study.
Field-collected samples	No field collected samples were used in the study.
Ethics oversight	No ethical approval is required for Caenorhabditis elegans, Caenorhabditis remanei and Drosophila melanogaster study.<br>We provided Ethics statement, "The animal work in this study was approved by the Nagoya University Animal Experiment Committee and was conducted in accordance with the “Regulations on Animal Experiments in Nagoya University” and “Guidelines for Proper Conduct of Animal Experiments (Science Council of Japan)”

Note that full information on the approval of the study protocol must also be provided in the manuscript.
