# Supplementary material for: Maintenance of quiescent oocytes by noradrenergic signals
Source: Nat Commun. 2021 Nov 26;12:6925. doi: 10.1038/s41467-021-26945-x (PMC8626438; doi:10.1038/s41467-021-26945-x)
Supplement: Supplementary file 3 — Description of Additional Supplementary Files [file 41467_2021_26945_MOESM3_ESM.pdf]

Description of Additional Supplementary Files

Supplementary Movie 1: wild type ovulation

Supplementary Movie 2: *tbh-1* mutant's ovulation is normal.

Supplementary Movie 3: *tdc-1* mutant's ovulation is normal.

Each video is running 10 times of the recorded speed.
